# Down-regulation of Irf8 by Lyz2-cre/loxP accelerates osteoclast differentiation in vitro

**DOI:** 10.1007/s10616-016-0013-z

**Published:** 2016-08-08

**Authors:** Emi Saito, Dai Suzuki, Daisuke Kurotaki, Ayako Mochizuki, Yoko Manome, Tetsuo Suzawa, Yoichi Toyoshima, Takahiro Ichikawa, Takahiro Funatsu, Tomio Inoue, Masamichi Takami, Tomohiko Tamura, Katsunori Inagaki, Ryutaro Kamijo

**Affiliations:** 1 Departments of Biochemistry, School of Dentistry, Showa University, 1-5-8 Hatanodai, Shinagawa, Tokyo 142-8555 Japan; 2 Department of Orthopedic Surgery, School of Medicine, Showa University, 1-5-8 Hatanodai, Shinagawa, Tokyo 142-8555 Japan; 3 Department of Immunology, Graduate School of Medicine, Yokohama City University, 3-9 Fukuura, Kanazawa-ku, Yokohama, Kanagawa 236-0004 Japan; 4 Department of Oral Physiology, School of Dentistry, Showa University, 1-5-8 Hatanodai, Shinagawa, Tokyo 142-8555 Japan; 5 Division of Dentistry for Persons with Disabilities, Department of Special Needs Dentistry, Showa University Dental Hospital, 2-1-1 Kitasenzoku, Ota, Tokyo 145-8515 Japan; 6 Department of Pharmacology, School of Dentistry, Showa University, 1-5-8 Hatanodai, Shinagawa, Tokyo 142-8555 Japan

**Keywords:** BMMs, Irf8, Lysozyme M, Lyz2, Osteoclasts

## Abstract

Interferon regulatory factor 8 (Irf8) is a transcription factor that negatively regulates osteoclast differentiation and Irf8 global knockout (*Irf8* ^−/−^) mice have been shown to have reduced bone volume resulting from increased osteoclast numbers. However, detailed analysis of the functions of Irf8 in osteoclast precursors with a monocyte/macrophage linage is difficult, because the population and properties of hematopoietic cells in *Irf8* ^−/−^ mice are severely altered. Therefore, to clearly elucidate the functions of Irf8 during osteoclastogenesis, we established myeloid cell-specific Irf8 conditional knockout (*Irf8* ^*fl/fl*^ *;Lyz2* ^*cre/*+^) mice. We found that trabecular bone volume in the *Irf8* ^*fl/fl*^ *;Lyz2* ^*cre/*+^ mice was not significantly affected, while exposure to M-CSF and RANKL significantly increased TRAP activity in vitro in osteoclasts that underwent osteoclastogenesis from bone marrow-derived macrophages (BMMs) induced from bone marrow cells (BMCs) of those mice by addition of M-CSF. Our results also showed that expression of Irf8 mRNA and protein in BMMs obtained from *Irf8* ^*fl/fl*^ *;Lyz2* ^*cre/*+^ mice and cultured with M-CSF was reduced. These findings predicted that Lyz2/Lyz2-cre expression is induced when BMCs differentiate into BMMs in cultures with M-CSF. In osteoclast differentiation cultures, *Lyz2* was gradually increased by M-CSF during the first 3 days of culture, then rapidly decreased by the addition of RANKL with M-CSF during the next 3 days. Furthermore, BMCs differentiated into osteoclasts while maintaining a low level of *Lyz2* expression when cultured simultaneously with both M-CSF and RANKL from the initiation of culture. These findings suggest that Lyz2-cre expression is induced along with differentiation to BMMs by BMCs obtained from *Irf8* ^*fl/fl*^ *;Lyz2* ^*cre/*+^ mice and cultured with M-CSF. In addition, Irf8 was down-regulated by activation of the cre/loxP recombination system in BMMs and osteoclastogenesis was accelerated. Based on our results, we propose the existence in vivo of a new lineage of osteoclast precursors among BMCs, which differentiate into osteoclasts without up-regulation of Lyz2 expression.

**Electronic supplementary material:**

The online version of this article (doi:10.1007/s10616-016-0013-z) contains supplementary material, which is available to authorized users.

## Introduction

The strength and health of bone tissues are regulated by a tight balance of bone resorption by osteoclasts and bone formation by osteoblasts. When that balance is destructed along with enhanced osteoclast and/or failed osteoblast function, bone develops osteopenia or osteoporosis, which is associated with a high risk of fracture. Osteoclasts are differentiated from osteoclast precursors with a monocyte/macrophage lineage following stimulation with macrophage colony-stimulating factor (M-CSF) and receptor activator of nuclear factor-κB ligand (RANKL), which are secreted from osteoblasts and osteocytes (Kobayashi et al. 2009; Nakashima et al. 2012). Osteoclast differentiation and activation are strictly controlled by various transcription factors, including AP-1 (Fos/Jun) and nuclear factor of activated T cells c1 (NFATc1), which are activated by RANKL signaling. Differentiated osteoclasts express several specific markers, including tartrate-resistant acid phosphatase (TRAP) and cathepsin K, a cysteine protease.

We previously reported that interferon regulatory factor 8 (IRF8), a transcription factor otherwise known as interferon consensus sequence binding protein (ICSBP), negatively regulates osteoclast differentiation (Zhao et al. 2009). Irf8 is expressed in osteoclast precursors and inhibits Nfatc1 function, and is down-regulated by stimulation with RANKL, which leads to Nfatc1 auto-amplification and osteoclast differentiation. Furthermore, Irf8 global knockout (*Irf8* ^−/−^) mice show reduced bone volume as a result of increased numbers of osteoclasts.

The population and properties of hematopoietic cells in *Irf8* ^−/−^ mouse bone marrow and spleen tissues are dramatically changed, along with development of chronic myelogenous leukemia and splenomegaly (Holtschke et al. 1996; Tamura et al. 2015; Tamura and Ozato 2002), thus it is difficult to clearly analyze the functions of Irf8 in monocytes/macrophages in the osteoclast precursor stage. In the present study, to investigate the detailed functions of Irf8 in monocytes/macrophages during osoteoclastogenesis, we established myeloid cell-specific Irf8 conditional knockout (*Irf8* ^*fl/fl*^ *;Lyz2* ^*cre/*+^) mice by crossing Irf8-flox with Lyz2-cre mice, and analyzed bone phenotype and osteoclast differentiation.

## Materials and methods

### Generation of mice

All animal experiments were conducted in accordance with the guidelines of Showa University. The Irf8 knockout (*Irf8* ^−/−^) mice (C57BL/6) used in this study have been described (Holtschke et al. 1996). Myeloid cell-specific Irf8 conditional knockout (*Irf8* ^*fl/fl*^ *;Lyz2* ^*cre/*+^) mice (C57BL/6) were generated by mating Irf8-flox with Lyz2-cre knock-in mice (Clausen et al. 1999; Feng et al. 2011). The primers used for genotyping are shown in Table S1.

### X-ray micro-tomography

Following euthanasia, tibiae were dissected and subjected to three-dimensional micro-computed tomography (μCT) with a ScanXmate-L090H (Comscan Tecno, Yokohama, Japan). Three-dimensional microstructural image data thus obtained were reconstructed using TRI/3D-BON software (Ratoc System Engineering, Tokyo, Japan).

### Cell cultures

Mouse bone marrow cells (BMCs) were collected from the femora and tibiae of 6- to 8-week-old male mice. Bone marrow-derived macrophages (BMMs) were formed from BMCs cultured in α-MEM supplemented with 10 % fetal bovine serum and M-CSF (50 ng/mL) for 3–4 days at 37 °C in a CO_2_ incubator (5 % CO_2_, 95 % air). Osteoclasts were formed from BMM (or BMC in Fig. 3c, d) cultures with M-CSF and various concentrations of RANKL after 3–4 days. Human M-CSF (Leucoprol) and RANKL was purchased from Kyowa Hakko Kogyo (Tokyo, Japan) and R&D Systems (Minneapolis, MN, USA), respectively.

### Detection of osteoclasts and measurement of TRAP activity

After culturing, cells were fixed with formalin and stained for tartrate-resistant acid phosphatase (TRAP; osteoclast marker) using a conventional method with naphthol AS-MX phosphate (Sigma-Aldrich, St. Louis, MO, USA) and fast red violet LB salt (Sigma-Aldrich) dissolved in 0.1 M acetic buffer (pH 5.0) containing 1 % tartrate acid (Suda et al. 1997). To evaluate the generation of osteoclasts, we used a TRAP activity assay, as previously described (Mochizuki et al. 2006).

### Western blot analysis

Cells were lysed in RIPA buffer and quantified using a BCA Protein Assay Kit (Thermo Scientific, Waltham, MA, USA). SDS-polyacrylamide gel electrophoresis sample buffer was added to protein samples (30 μg), followed by boiling for 5 min. Next, the samples were loaded onto pre-cast gradient Mini-PROTEAN TGX Gels (Bio-Rad Laboratories, Hercules, CA, USA), then separated and transferred to PVDF Immobilon-P membranes (Merck Millipore, Billerica, MA, USA) using a Mini Trans-Blot Cell system (Bio-Rad Laboratories). The membranes were blocked in 5 % BSA Tris-buffered saline-Tween 20, incubated with primary antibodies according to the supplier’s instructions, and incubated with appropriate HRP-conjugated secondary antibodies prior to signal detection with SuperSignal West Substrate (Thermo Scientific) using a VersaDoc Imaging System (Bio-Rad Laboratories). The antibodies used in the present experiment were as follows: anti-Irf8 antibody (Icsbp) #sc6058 (Santa Cruz Biotechnology, Santa Cruz, CA, USA), anti-β-actin antibody #A5060 (Sigma-Aldrich), anti-goat IgG-HRP #sc2768 (Santa Cruz Biotechnology), and anti-rabbit IgG-HRP #7074 (Cell Signaling Technology, Danvers, MA, USA).

### Quantitative real-time PCR

Total RNA was extracted with TRIzol reagent (Invitrogen, Carlsbad, CA, USA), then reverse-transcribed using ReverTra Ace qPCR RT Master Mix (TOYOBO, Osaka, Japan). Quantitative real-time PCR (qPCR) was performed using THUNDERBIRD SYBR qPCR Mix (TOYOBO) and the StepOne Real-Time PCR System (Applied Biosystems, Foster City, CA, USA). The primer sequences were as follows: *Actb*, 5′-AGATGACCCAGATCATGTTTGAGA-3′ and 5′-CACAGCCTGGATGGCTACGT-3′; *Irf8*, 5′-GGTGGATGCTTCCATCTTCAA-3′ and 5′-GTGGCTGGTTCAGCTTTGTCT-3′; *Ctsk*, 5′-CGACTATCGAAAGAAAGGATACGTT-3′ and 5′-AGCCCAACAGGAACCACACT-3′; and *Lyz2*, 5′-AATGGCTGGCTACTATGGAGTCA-3′ and 5′-TGCTCTCGTGCTGAGCTAAACA-3′. Expressions were normalized to that of *Actb*.

### Statistical analysis

The results are expressed as the mean ± SD for each experiment. Student’s two-tailed *t* test was used, with *p* < 0.05 considered to indicate significance.

## Results

**Fig. 1:**
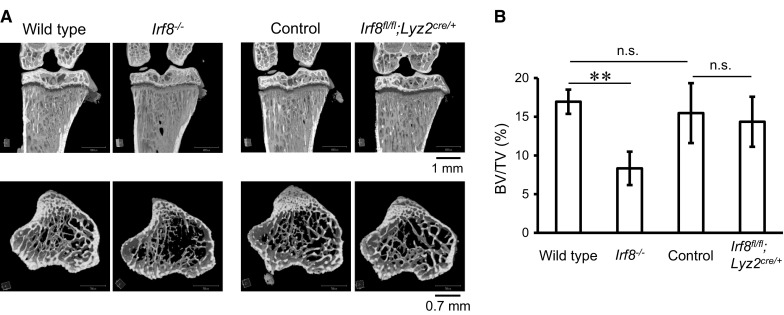
*Irf8* ^*fl/fl*^ *;Lyz2* ^*cre/*+^ mice did not develop osteoporosis. **a** Representative μCT images of vertical (*upper panels*) and axial (*lower panels*) tibiae obtained from 8-week-old male mice of the indicated genotypes. **b** μCT analysis of bone volume per tissue volume (BV/TV) of tibia trabecular bone (wild-type, *n* = 8; *Irf8* ^−/−^, n = 6; control, n = 20; *Irf8* ^*fl/fl*^ *;Lyz2* ^*cre/*+^, n = 23). **p < 0.01; *n.s.* not significant

We initially evaluated the bone morphology of *Irf8* ^*fl/fl*^ *;Lyz2* ^*cre/*+^ mice, for which tibiae from proximal sides of 8-week-old males were analyzed using μCT. As we previously reported, trabecular bone in *Irf8* ^−/−^ mice was found to be sparse (Fig. 1a) and bone volume per tissue volume (BV/TV) of tibia trabecular bone in those mice was reduced by approximately 50 % as compared to that of wild-type mice (Fig. 1b). On the other hand, as compared to the control group (*Irf8* ^*fl/fl*^ or *Irf8* ^*fl/*+^ *;Lyz2* ^*cre/*+^), trabecular bone in the *Irf8* ^*fl/fl*^ *;Lyz2* ^*cre/*+^ mice was not altered (Fig. 1a) and BV/TV was not significantly changed (Fig. 1b). Furthermore, there were no significant difference in regard to BV/TV between the control group and wild-type mice. In addition, the body weights of the *Irf8* ^*fl/fl*^ *;Lyz2* ^*cre/*+^ mice during development were not different as compared to the control mice, while splenomegaly was observed in the *Irf8* ^−/−^ but not the *Irf8* ^*fl/fl*^ *;Lyz2* ^*cre/*+^ mice (data not shown). These results suggest that osteoclastogenesis in bone tissues of *Irf8* ^*fl/fl*^ *;Lyz2* ^*cre/*+^ mice is not enhanced in vivo.

**Fig. 2:**
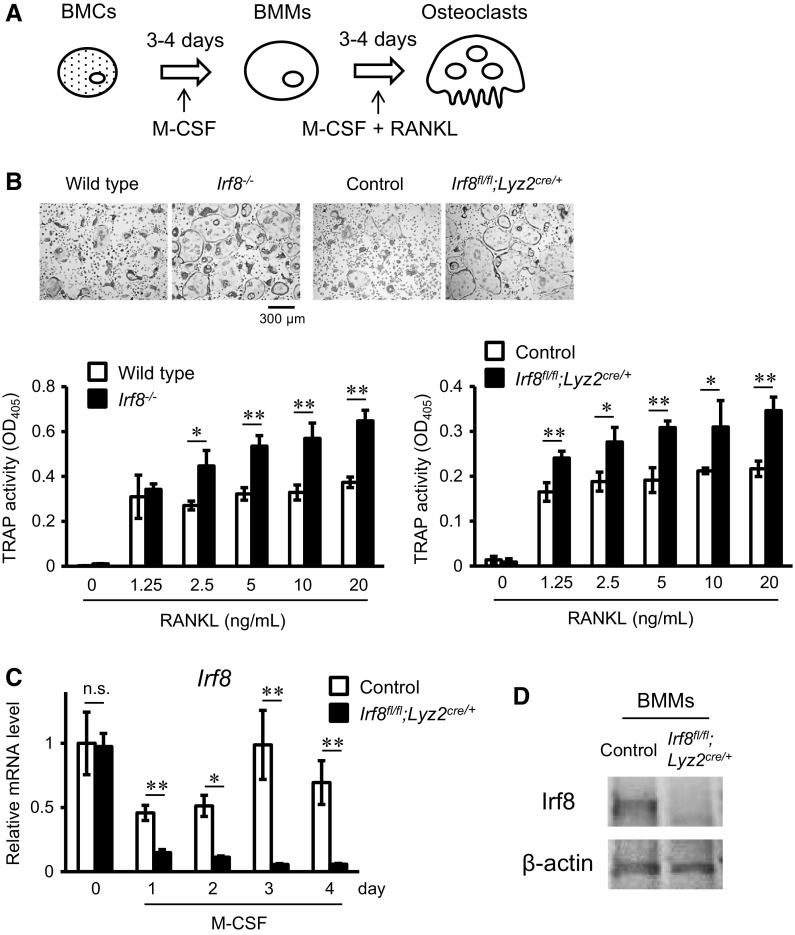
Osteoclastogenesis induced in vitro by M-CSF and RANKL was enhanced in cultures of BMMs obtained from *Irf8* ^*fl/fl*^ *;Lyz2* ^*cre/*+^ mice. **a** Schema of conventional osteoclast culture system. **b** Representative TRAP staining of osteoclasts differentiated from BMMs cultured with M-CSF and RANKL (20 ng/mL) for the indicated genotypes (*upper panels*). Shown is TRAP activity of osteoclasts differentiated from BMMs cultured with M-CSF and the indicated doses of RANKL (*lower graphs*). **c** qPCR analysis of *Irf8* mRNA expression in BMCs from control and *Irf8* ^*fl/fl*^ *;Lyz2* ^*cre/*+^ mice cultured with M-CSF for 0–4 days. **d** Western blot analysis of Irf8 and β-actin in BMMs differentiated from BMCs after 3 days of culture with M-CSF. *p < 0.05, **p < 0.01; *n.s.* not significant

Next, to investigate the potential of *Irf8* ^*fl/fl*^ *;Lyz2* ^*cre/*+^ mouse BMMs to differentiate into osteoclasts in vitro, we used a conventional osteoclast culture system, in which BMCs were induced to differentiate into BMMs by M-CSF, then the BMMs were differentiated into osteoclasts by addition of M-CSF and RANKL (Fig. 2a). We found that osteoclast formation by BMMs from *Irf8* ^*fl/fl*^ *;Lyz2* ^*cre/*+^ mice was enhanced as compared to the control group, while that by BMMs from *Irf8* ^−/−^ mice was enhanced as compared to the wild-type mice (Fig. 2b, upper panels). Furthermore, TRAP activity in osteoclasts from the *Irf8* ^*fl/fl*^ *;Lyz2* ^*cre/*+^ mice was significantly increased as compared to the control, while alterations in the *Irf8* ^−/−^ and wild-type mice were similar (Fig. 2b, lower graphs). Using this culture system, we also evaluated mRNA and protein expression levels of Irf8 using qPCR and western blotting, respectively. Our results showed that *Irf8* mRNA expression in BMCs was not significantly different between the control and *Irf8* ^*fl/fl*^ *;Lyz2* ^*cre/*+^ mice (Fig. 2c, day 0), while that in BMMs from *Irf8* ^*fl/fl*^ *;Lyz2* ^*cre/*+^ mice induced by M-CSF was significantly suppressed as compared to the control group (Fig. 2c, days 1–4). In addition, the Irf8 protein expression level in BMMs induced from BMCs of *Irf8* ^*fl/fl*^ *;Lyz2* ^*cre/*+^ mice after 3 days of culture with M-CSF was decreased as compared to the control mice (Fig. 2d). Together, these findings suggest that the progression of differentiation of *Irf8* ^*fl/fl*^ *;Lyz2* ^*cre/*+^ mouse BMMs to osteoclasts in vitro is induced by suppression of Irf8 expression in BMMs caused by the presence of M-CSF in the culture.

**Fig. 3:**
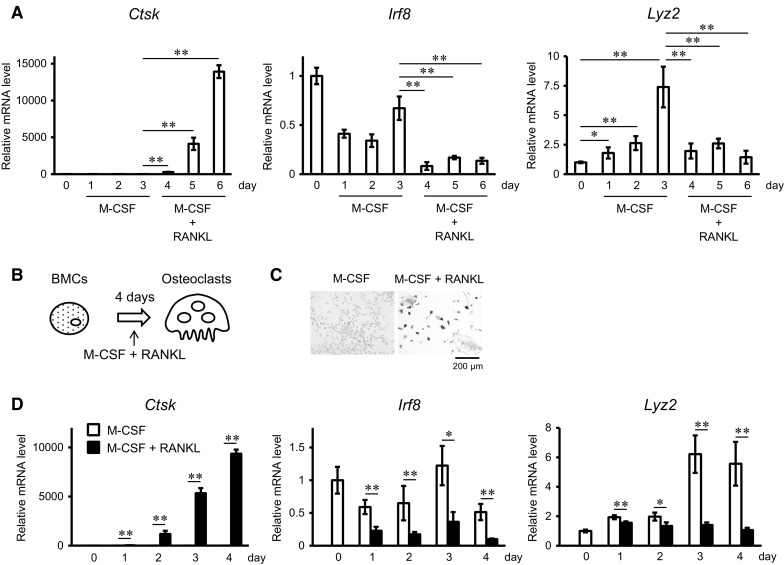
*Lyz2* is up-regulated by M-CSF and down-regulated by RANKL in BMC and BMM cultures. **a** qPCR analysis of *Ctsk*, *Irf8*, and *Lyz2* mRNA expressions in BMCs from wild-type mice cultured with M-CSF for days 0–3, then with M-CSF and RANKL (100 ng/mL) for days 4–6. **b** Schema of direct osteoclast culture system using BMCs. **c** Representative TRAP staining of osteoclasts differentiated from BMCs cultured with only M-CSF, or simultaneously with both M-CSF and RANKL (100 ng/mL) from the initiation of culture. **d** qPCR analysis of *Ctsk*, *Irf8*, and *Lyz2* mRNA expressions in BMCs from wild-type mice cultured with only M-CSF, or simultaneously with both M-CSF and RANKL (100 ng/mL) for 4 days from the initiation of culture (*lower panel*). *p < 0.05, **p < 0.01

Our results predicted that Lyz2/Lyz2-cre expression is induced when BMCs differentiate into BMMs in cultures with M-CSF, using a conventional osteoclast culture system, thus we investigated the fluctuation of *Lyz2* mRNA expression using qPCR. Those results confirmed that the expression of *Ctsk* (encodes Cathepsin K; osteoclast marker) was greatly increased, while that of *Irf8* was reduced by stimulation with RANKL (Fig. 3a, days 4–6). Therefore, we found that the expression of *Lyz2* was gradually increased by M-CSF (Fig. 3a, days 1–3) and rapidly decreased by RANKL stimulation (Fig. 3a, days 4–6). Next, to examine whether osteoclast precursors among BMCs are able to differentiate into osteoclasts without the increased expression of *Lyz2* seen in M-CSF-cultured BMMs, we compared BMC cultures between those stimulated with only M-CSF and those simultaneously stimulated with both M-CSF and RANKL from the initiation of culture (Fig. 3b). Those finding showed that TRAP-positive osteoclasts were formed from cultures of BMCs stimulated by both M-CSF and RANKL (Fig. 3c), as well as dramatic time-dependent up-regulation of *Ctsk* expression in BMCs cultured under that condition (Fig. 3d). Also, BMCs stimulated simultaneously with both M-CSF and RANKL maintained low expression levels of *Irf8* and *Lyz2* as compared to those cultured with only M-CSF (Fig. 3d). These results indicate that osteoclast precursors among BMCs are able to differentiate into osteoclasts without up-regulation of *Lyz2* expression when in the presence of both M-CSF and RANKL from the initiation of culture.

## Discussion

**Fig. 4:**
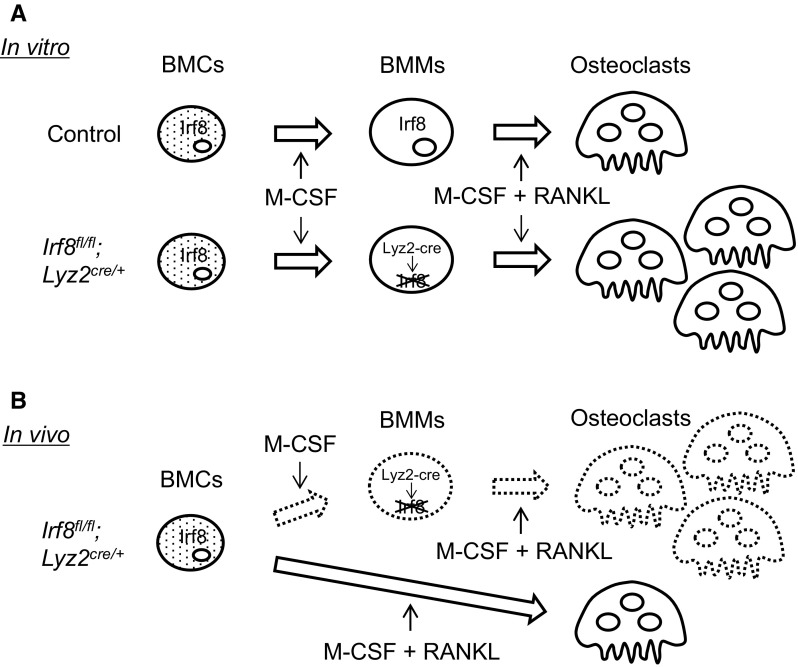
Schematic representation of osteoclastogenesis of *Irf8* ^*fl/fl*^ *;Lyz2* ^*cre/*+^ mouse BMCs in vitro and in vivo. **a** Lyz2-cre was induced along with differentiation of BMCs from *Irf8* ^*fl/fl*^ *;Lyz2* ^*cre/*+^ mice into BMMs in cultures with M-CSF. Irf8 in the BMMs was then deleted by activation of the cre/loxP recombination system and osteoclastogenesis was accelerated. **b** Proposed novel in vivo differentiation lineage, in which osteoclast precursors among BMCs differentiate into osteoclasts by simultaneous exposure to M-CSF and RANKL, and do not show Lyz2/Lyz2-cre expression. This differentiation lineage may be dominant in vivo and could explain why *Irf8* ^*fl/fl*^ *;Lyz2* ^*cre/*+^ mice do not develop osteoporosis

In contrast to *Irf8* ^−/−^ mice, the *Irf8* ^*fl/fl*^ *;Lyz2* ^*cre/*+^ mice did not develop osteoporosis. However, BMMs from the *Irf8* ^*fl/fl*^ *;Lyz2* ^*cre/*+^ mice with a low level of Irf8 expression induced by M-CSF from BMCs in vitro aggressively differentiated into osteoclasts by RANKL stimulation, similar to those from the *Irf8* ^−/−^ mice. We also noted that *Lyz2* expression was up-regulated in cultures with M-CSF, namely, *Lyz2* expression in BMMs was higher than that in BMCs. These results suggested that Lyz2-cre was induced along with the differentiation of BMCs from *Irf8* ^*fl/fl*^ *;Lyz2* ^*cre/*+^ mice to BMMs when exposed to M-CSF. Thereafter, loxP-flanked *Irf8* DNA was deleted by activation of the cre/loxP recombination system in BMMs from *Irf8* ^*fl/fl*^ *;Lyz2* ^*cre/*+^ mice and osteoclastogenesis induced by RANKL was accelerated (Fig. 4a).

On the other hand, this is the first study to show that osteoclast precursors among BMCs differentiated into osteoclasts while maintaining a low level of *Lyz2* expression when simultaneously exposed to both M-CSF and RANKL from the initiation of culture. That finding raises the possibility of another osteoclast lineage that differentiates from osteoclast precursors among BMCs and does not express Lyz2/Lyz2-cre, which may be dominant in vivo as compared to osteoclasts with BMM lineage (Fig. 4b). *Irf8* mRNA expression was not reduced in BMCs from *Irf8* ^*fl/fl*^ *;Lyz2* ^*cre/*+^ mice and those mice did not demonstrate osteoporosis. Thus, if most osteoclasts differentiate from BMMs in vivo, then *Irf8* ^*fl/fl*^ *;Lyz2* ^*cre/*+^ mice should develop osteoporosis caused by enhancement of excessive bone resorption induced by osteoclastogenesis, the same as seen in *Irf8* ^−/−^ mice. However, that does not occur in *Irf8* ^*fl/fl*^ *;Lyz2* ^*cre/*+^ mice. A previous study showed that mice obtained by crossing Nfatc1 conditional knockout mice with Lyz2-cre mice had no alterations in bone density (Aliprantis et al. 2008). Nfatc1 is a master regulator of osteoclast differentiation and activated by RANKL, and its transcriptional activity and expression are inhibited by Irf8 in osteoclast precursors (Zhao et al. 2009). In other words, the main portion of osteoclasts in vivo is the results of differentiation from osteoclast precursors under regulations of Irf8 and Nfatc1, without Lyz2/Lyz2-cre expression. However, Lyz2-cre mice have been used in osteoclast differentiation studies, and conditional knockout mice have been found to have such bone phenotypes as osteoporosis (Albers et al. 2013; Martin-Millan et al. 2010) and osteopetrosis (Kenner et al. 2004; Wang et al. 2008). Thus, the process of osteoclast differentiation may be dependent on a combination of between expression level and/or function of each regulator, and two types of osteoclast precursors, those with and without Lyz2/Lyz2-cre expression. Additional studies are needed to elucidate the mechanisms involved.

## Electronic supplementary material

Below is the link to the electronic supplementary material.

Supplemental Table 1. Primer sequences for genomic PCR analysis. Irf8 knockout, Irf8-flox, and Lyz2-cre knock-in mice were genotyped using the PCR primer sets. Offspring were identified by the indicated PCR product sizes. (PPTX 52 kb)

## References

[CR1] Albers J (2013). Canonical Wnt signaling inhibits osteoclastogenesis independent of osteoprotegerin. J Cell Biol.

[CR2] Aliprantis AO (2008). NFATc1 in mice represses osteoprotegerin during osteoclastogenesis and dissociates systemic osteopenia from inflammation in cherubism. J Clin Invest.

[CR3] Clausen BE, Burkhardt C, Reith W, Renkawitz R, Forster I (1999). Conditional gene targeting in macrophages and granulocytes using LysMcre mice. Transgenic Res.

[CR4] Feng J, Wang H, Shin DM, Masiuk M, Qi CF, Morse HC (2011). IFN regulatory factor 8 restricts the size of the marginal zone and follicular B cell pools. J Immunol.

[CR5] Holtschke T (1996). Immunodeficiency and chronic myelogenous leukemia-like syndrome in mice with a targeted mutation of the ICSBP gene. Cell.

[CR6] Kenner L (2004). Mice lacking JunB are osteopenic due to cell-autonomous osteoblast and osteoclast defects. J Cell Biol.

[CR7] Kobayashi Y, Udagawa N, Takahashi N (2009). Action of RANKL and OPG for osteoclastogenesis. Crit Rev Eukaryot Gene Expr.

[CR8] Martin-Millan M (2010). The estrogen receptor-alpha in osteoclasts mediates the protective effects of estrogens on cancellous but not cortical bone. Mol Endocrinol.

[CR9] Mochizuki A (2006). Identification and characterization of the precursors committed to osteoclasts induced by TNF-related activation-induced cytokine/receptor activator of NF-kappa B ligand. J Immunol.

[CR10] Nakashima T, Hayashi M, Takayanagi H (2012). New insights into osteoclastogenic signaling mechanisms. Trends Endocrinol Metab.

[CR11] Suda T, Jimi E, Nakamura I, Takahashi N (1997). Role of 1 alpha,25-dihydroxyvitamin D3 in osteoclast differentiation and function. Methods Enzymol.

[CR12] Tamura T, Ozato K (2002). ICSBP/IRF-8: its regulatory roles in the development of myeloid cells. J Interf Cytokine Res.

[CR13] Tamura T, Kurotaki D, Koizumi S (2015). Regulation of myelopoiesis by the transcription factor IRF8. Int J Hematol.

[CR14] Wang Y, Lebowitz D, Sun C, Thang H, Grynpas MD, Glogauer M (2008). Identifying the relative contributions of Rac1 and Rac2 to osteoclastogenesis. J Bone Miner Res.

[CR15] Zhao B (2009). Interferon regulatory factor-8 regulates bone metabolism by suppressing osteoclastogenesis. Nat Med.

