# Supplementary material for: Down-regulation of Irf8 by Lyz2-cre/loxP accelerates osteoclast differentiation in vitro
Source: Cytotechnology. 2016 Aug 8;69(3):443–50. doi: 10.1007/s10616-016-0013-z (PMC5461233; doi:10.1007/s10616-016-0013-z)
Supplement: Supplementary file 1 — Supplemental Table 1. Primer sequences for genomic PCR analysis. Irf8 knockout, Irf8-flox, and Lyz2-cre knock-in mice were genotyped using the PCR primer sets. Offspring were identified by the indicated PCR product sizes. (PPTX 52 kb) [file 10616_2016_13_MOESM1_ESM.pptx]

## Slide 1
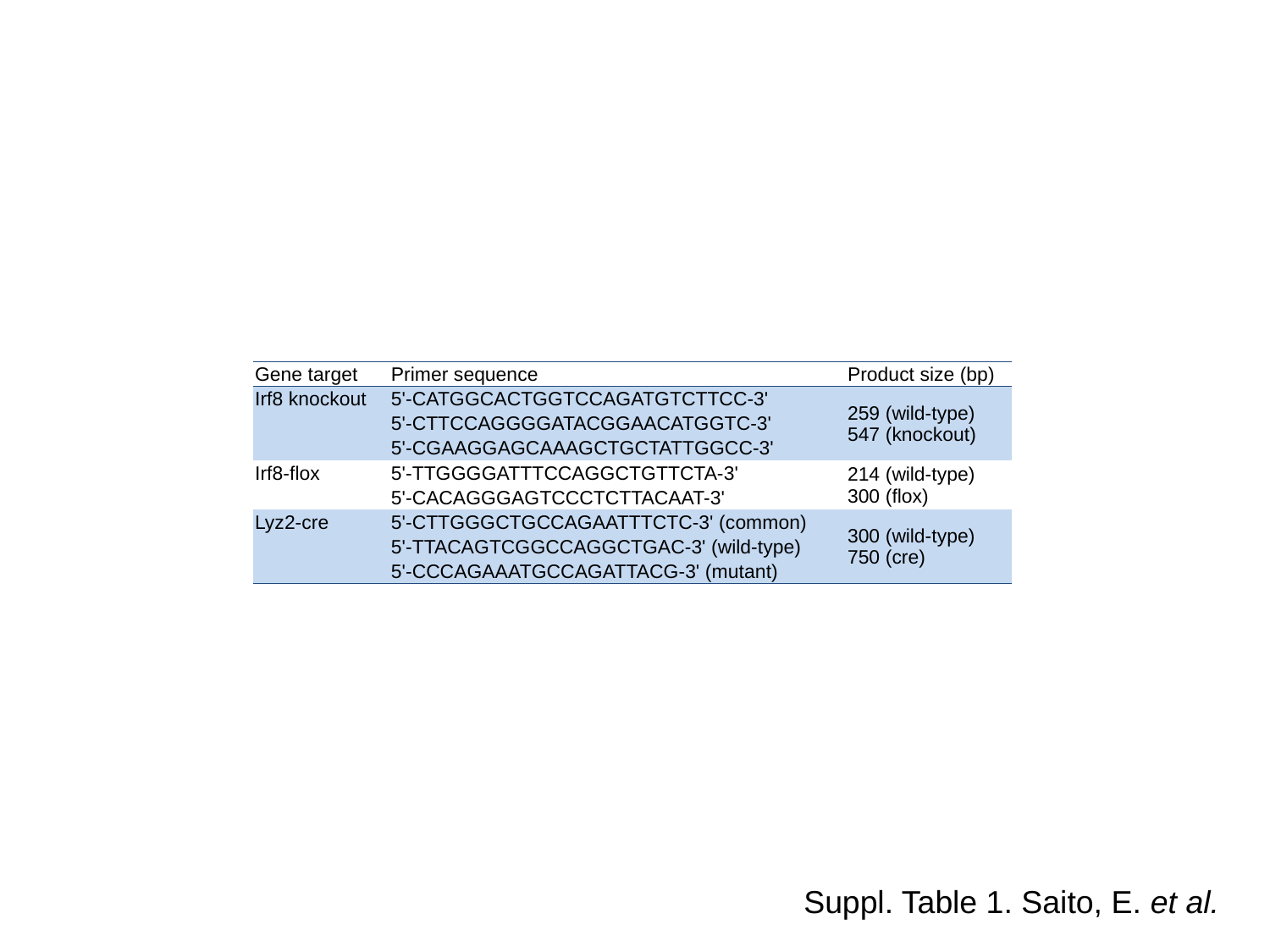

| Gene target | Primer sequence | Product size (bp) |
| --- | --- | --- |
| Irf8 knockout | 5'-CATGGCACTGGTCCAGATGTCTTCC-3' | 259 (wild-type) 547 (knockout) |
| | 5'-CTTCCAGGGGATACGGAACATGGTC-3' | |
| | 5'-CGAAGGAGCAAAGCTGCTATTGGCC-3' | |
| Irf8-flox | 5'-TTGGGGATTTCCAGGCTGTTCTA-3' | 214 (wild-type) 300 (flox) |
| | 5'-CACAGGGAGTCCCTCTTACAAT-3' | |
| Lyz2-cre | 5'-CTTGGGCTGCCAGAATTTCTC-3' (common) | 300 (wild-type) 750 (cre) |
| | 5'-TTACAGTCGGCCAGGCTGAC-3' (wild-type) | |
| | 5'-CCCAGAAATGCCAGATTACG-3' (mutant) | |
Suppl. Table 1. Saito, E. et al.
